# Unequal Burden: Racial Differences in Mortality, Complications, and Resource Utilization in Immune Thrombocytopenia Hospitalizations

**DOI:** 10.7759/cureus.87242

**Published:** 2025-07-03

**Authors:** Tijin Mathew, Teresa M Varghese, George M Varghese

**Affiliations:** 1 Internal Medicine, Southeast Health Medical Center, Dothan, USA; 2 Internal Medicine, Welllstar Spalding, Spalding, USA; 3 Cardiovascular Medicine, Stanford University, California, USA

**Keywords:** bleeding risk, idiopathic thrombocytopenic purpura, immune thrombocytopenia, immune thrombocytopenic purpura, racial disparity

## Abstract

Introduction

Immune thrombocytopenia (ITP) is characterized by the autoimmune destruction of platelets and is a diagnosis of exclusion. It can range from asymptomatic to spontaneous bleeding. Race significantly affects healthcare utilization and clinical outcomes of ITP hospitalizations. We aimed to determine the number of ITP hospitalizations in 2022 and investigate how race influences admission, clinical outcomes, and healthcare utilization.

Methods

We retrospectively analyzed ITP-related hospitalizations using the 2022 National Inpatient Sample (NIS). Patients were categorized by race into six groups: White, African American, Hispanic, Asian or Pacific Islander, Native American, and Other. Data analysis was conducted using STATA/BE version 18.5. Univariate and multivariable logistic regression models were used to assess the associations between race, clinical outcomes, and healthcare utilization metrics.

Results

In 2022, 315,277 hospitalizations were recorded for ITP. Of these, 56.35% were male and 43.65% were female. 64.5% of patients identified themselves as White, 12.5% as Hispanic, 13.3% as African-American, 3.5% as Asian or Pacific Islander, 0.9% as Native American, and 2.9% as other minority groups. The highest in-hospital mortality among ITP patients was observed in Asian or Pacific Islander individuals (8.83%), followed by African American (8.32%) and those categorized as Other (8.25%). Mortality rates were slightly lower in Native American (7.54%) and White (7.22%) patients, while Hispanic patients had the lowest rate (6.80%), with a p-value <0.001.

Conclusion

This study reveals significant racial and socio-economic disparities in hospitalization outcomes among patients with ITP. Minority populations, particularly Asian or Pacific Islander, African American, and Native American patients, experienced higher mortality rates. These findings highlight the urgent need for targeted interventions, inclusive clinical research, and policy reforms to address social determinants of health and ensure equitable care and outcomes for all individuals affected by ITP.

## Introduction

Immune thrombocytopenia (ITP), also known as idiopathic or immune thrombocytopenic purpura, is a hematological disease characterized by the autoimmune destruction of platelets [1]. As per the American Society of Hematology 2019 guideline for ITP, it's a diagnosis of exclusion [2]. This clinical condition is characterized by a low platelet count of less than 100,000 per microliter without other causes of thrombocytopenia, and the bleeding risk is not correlated with the degree of platelet count [3,4]. ITP can range from asymptomatic to spontaneous bleeding, including life-threatening bleeding, such as intracerebral hemorrhage [5].

Racial disparities in the outcomes of ITP have been increasingly recognized in recent years, and recent Nationwide data from the United States demonstrated that, although there is a decrease in overall mortality among hospitalized ITP patients over the past decade, this was observed exclusively in the White population [6]. However, Black and Hispanic patients have not experienced a similar reduction in mortality, which highlights racial disparities in the outcomes of ITP [6]. Our study aimed to identify the total ITP hospitalizations in 2022 from the National Inpatient Sample (NIS) and to understand how race affects admission, mortality, and healthcare utilization. 

## Materials and methods

Database: NIS 2022

This research utilized the NIS, a well-established and publicly available database in the United States that compiles inpatient data from various healthcare payers. The NIS serves as a valuable tool for large-scale analysis, providing comprehensive insights into hospital usage, patient demographics, healthcare expenditures, insurance coverage, quality measures, and clinical outcomes at both regional and national levels. The dataset is developed through the Healthcare Cost and Utilization Project (HCUP), a collaborative initiative by the Agency for Healthcare Research and Quality (AHRQ) and supported by federal, state, and private organizations. It employs a stratified sampling approach that captures approximately 20% of all annual hospital discharges in the United States. In accordance with Southeast Health's institutional policies, studies using completely de-identified datasets like the NIS are considered exempt from Institutional Review Board (IRB) oversight. Therefore, this study did not require IRB approval.

Study population and study variable

Data from the 2022 NIS were analyzed to identify adult hospitalizations (patients over 18 years) with a primary or secondary diagnosis of ITP, based on International Classification of Diseases, Tenth Revision, Clinical Modification (ICD-10-CM) codes. The ICD-10 codes used to identify the patient population include D69.3, D69.6, and D69.59. Patients' races were categorized as White, African American, Hispanic, Asian or Pacific Islander, Native American, or Other. The "Other" category typically includes individuals identifying as multiracial, those belonging to smaller racial groups not separately listed, or patients whose race was documented as "other" in hospital records. The study examined socio-demographic variables, such as age, sex, insurance type, income quartile, and hospital characteristics, across these racial groups, including facility size, geographic setting, United States's census region, and teaching status. The primary clinical outcomes evaluated were in-hospital mortality, discharge disposition, total hospitalization costs, and length of stay.

Statistical analysis

This study employed a cross-sectional design, and all statistical analyses were performed using STATA/BE version 18.5. Associations between categorical variables were assessed using chi-square tests, while ANOVA was utilized to compare continuous variables. To evaluate differences in mean values in categorical variables, such as age, total charge, and length of stay, across racial groups, we used one-way ANOVA. As part of the ANOVA output, Bartlett’s test for equal variances was conducted, and the resulting chi-square statistic was used to evaluate the assumption of homogeneity of variances across groups. A p-value of less than 0.05 was considered indicative of statistical significance. Multivariable logistic regression models were constructed to examine the association between race and various clinical and healthcare utilization outcomes. These outcomes included admission type, discharge disposition, resource utilization, and complications such as epistaxis, platelet transfusions, acute kidney injury (AKI), and sepsis. The models were adjusted for potential confounders, including obesity, socio-demographic variables, and hospital-related characteristics.

## Results

Patient and hospital-level characteristics

In 2022, 315,277 hospitalizations were recorded for ITP. Of these, 56.35% were male and 43.65% were female. 64.5% of patients identified themselves as White, 12.5% as Hispanic, 13.3% as African-American, 3.5% as Asian or Pacific Islander, and 0.9% as Native American or 2.9% of other minority groups.

The average age of the patient population was 62 years, with a 95% confidence interval (CI) ranging from 62.6 to 62.7. The mean age of hospitalized patients with ITP varied significantly across racial groups (p < 0.001). White patients had the highest average age at 65 years, followed by Asian or Pacific Islander patients at 63 years. In contrast, Hispanic (55 years), Native American (54 years), African American (58 years), and Other racial groups (58 years) presented at significantly younger ages.

Gender distribution among patients hospitalized with ITP showed notable variation across racial groups, with differences reaching statistical significance (p < 0.001). Males accounted for the majority of hospitalizations overall, particularly among White (57.7%), Hispanic (55.9%), and Other (56.0%) racial groups. In contrast, female patients were slightly more represented among African American (48.6%), Asian or Pacific Islander (47.0%), and Native American (47.4%) groups. However, males still represent a slight majority in these categories. Income levels varied notably by race (p < 0.001), with over half of Native American and African American patients in the lowest quartile. In contrast, nearly half of Asian or Pacific Islander patients were in the highest income bracket.

Racial differences were evident in insurance type and hospital region among patients hospitalized with ITP (p < 0.001). White (62%) and Asian (51.7%) patients were predominantly covered by Medicare, reflecting older age demographics, while Medicaid coverage was highest among Native American (45%), Hispanic (34.4%), and African American (26%) patients, suggesting greater socio-economic vulnerability. Hispanic patients also had the highest self-pay rate (7.5%), indicating potential gaps in insurance access. Regionally, hospitalizations for White (37%) and African American (52.3%) patients were most common in the South, whereas Hispanic (40.2%), Asian or Pacific Islander (52.4%), and Native American (52.4%) patients were primarily hospitalized in the West. Patients categorized as “Other” were most frequently seen in the Northeast (33.1%), reflecting geographic variation in racial demographics and healthcare access. The patient and hospital-level characteristics of hospitalized ITP patients stratified by race in the NIS 2022 are summarized in Table 1.

**Table 1:** Patient and hospital-level characteristics of hospitalized ITP patients stratified by race in the 2022 NIS *: $1 - 55,999; **: $56,000 - 70,999; ***: $71,000 - 93,999; ****: $94,000 or more Chi-square (χ²) test was used to identify the above variables in each race. ANOVA test was used in the continuous variable like age. ITP: Immune thrombocytopenia; NIS: National Inpatient Sample

Variables	White (N=203,258) (%)	African-American (N=41,808) (%)	Hispanic (N=39,463) (%)	Asian or Pacific Islanders (N=11,054) (%)	Native American (N=2,722) (%)	Others (N=9,084) (%)	χ² (degrees of freedom)	p-Value
Age								
Mean Age (Years) ± SD	65 ± 17.5	58 ± 19.2	55 ± 20.0	63 ± 20.1	54 ± 17.2	58 ± 20.9	χ²(5) = 2.2 x 10^3^, F-value = 2730.6	<0.001
Gender								
Male (%)	57.67 (117,265)	51.36 (21,475)	55.93 (22,082)	52.96 (5,851)	52.63 (1,433)	56.02 (5,092)	χ²(5) = 637.76	<0.001
Female (%)	42.33 (85,993)	48.64 (20,333)	44.07 (17,381)	47.04 (5,203)	47.37 (1,289)	43.98 (3,992)		
Median Household Income National Quartiles								
Quartile 1 (0-25th percentile)*	23.13 (47,048)	48.76 (20,390)	34.53 (13,628)	11.22 (1,240)	51.89 (1,413)	21.94 (1,993)	χ²(15) = 1.9 × 10⁴	<0.001
Quartile 2 (26-50th percentile)**	26.08 (53,009)	21.86 (9,139)	25.22 (9,958)	17.32 (1,914)	23.72 (646)	21.27 (1,933)		
Quartile 3 (51-75th percentile)***	26.48 (53,816)	17.48 (7,309)	25.21 (9,954)	25.89 (2,860)	15.75 (429)	25.56 (2,324)		
Quartile 4 (76-100th percentile)****	24.31 (49,385)	11.9 (4,971)	15.04 (5,923)	45.57 (5,040)	8.64 (235)	31.23 (2,834)		
Insurance Type								
Medicare	62 (125,021)	50.62 (21,176)	38.59 (15,206)	51.7 (5,716)	35.54 (968)	44.09 (4,005)	χ²(15) = 1.8× 10⁴	<0.001
Medicaid	12.63 (25,677)	26.04 (10,894)	34.43 (13,589)	18.55 (2,051)	44.96 (1,224)	26.91 (2,444)		
Private including HMO	22.77 (46,306)	19.07 (7,972)	19.49 (7,689)	27.42 (3,030)	16.3 (444)	23.7 (2,152)		
Self-Pay	2.59 (5,254)	4.27 (1,785)	7.49 (2,956)	2.32 (257)	3.21 (87)	5.3 (482)		
Hospital-Region								
Northeast	18.69 (37,981)	16.51 (6,905)	15.64 (6,172)	19.37 (2,140)	3.75 (102)	33.12 (3,009)	χ²(5) = 2.9 x 10^4^	<0.001
Midwest	26.46 (53,785)	22.06 (9,215)	8.24 (3,252)	10.82 (1,196)	19.91 (542)	10.46 (951)		
South	37 (75,205)	52.30 (21,873)	35.88 (14,162)	17.41 (1,926)	23.99 (653)	30.7 (2,791)		
West	17.85 (36,287)	9.13 (3,815)	40.24 (15,837	52.41 (5,792)	52.35 (1,425)	25.72 (2,333)		
Bed Size								
Small	21.38 (43,433)	18.87 (7,890)	18.29 (7,216)	17.37 (1,918)	22.59 (615)	17.32 (1,574)	χ²(5) = 613.10	<0.001
Medium	27.84 (56,582)	26.51 (11,085)	27.87 (11,001)	24.85 (2,747)	26.3 (716)	27.28 (2,477)		
Large	50.78 (103,243)	54.63 (22,833)	53.84 (21,246)	57.87 (6,389)	51.10 (1,391)	55.41 (5,033)		

Primary outcomes

Among various racial groups, most patients were discharged home, with the highest proportions seen in Hispanic (58.09%) and Native American (57.37%) patients. Home healthcare was most common among Asian or Pacific Islander patients (22.59%), followed closely by White patients (20.42%). Discharge to a skilled nursing facility was most frequent among White patients (22.93%) and least common among Hispanic (12.76%) and Native American (15.56%) patients. The highest rate of discharge against medical advice occurred in Native American patients (3.79%). These differences were statistically significant (p < 0.001).

The highest in-hospital mortality among ITP patients was observed in Asian or Pacific Islander individuals (8.83%), followed by African American (8.32%) and those categorized as Other (8.25%). Mortality rates were slightly lower in Native American (7.54%) and White (7.22%) patients, while Hispanic patients had the lowest rate at 6.80%. These statistically significant differences (p < 0.001) may reflect underlying disparities in health status, access to timely care, or disease severity at presentation.

The average length of hospital stays for African American individuals and those classified as Other was the longest at nine days, while Hispanic patients had the shortest stays at seven days. Similarly, total hospital charges varied significantly, with the highest costs observed among the Other racial group ($178,797) and Asian or Pacific Islander patients ($162,031). The lowest average charges for Native American patients were $102,711 (p < 0.001). The racial inequities in admission and disposition outcomes and healthcare utilization among ITP patients are summarized in Table 2.

**Table 2:** Racial inequities in admission and disposition outcomes and healthcare utilization among ITP patients from 2022 NIS Chi-square (χ²) test was used to identify the above variables in each race. ANOVA test was used in continuous variable like length of stay and total charge. ITP: Immune thrombocytopenia; NIS: National Inpatient Sample

Outcomes	White (N=203,258) (%)	African-American (N=41,808) (%)	Hispanic (N=39,463) (%)	Asian or Pacific Islanders (N=11,054) (%)	Native American (N=2,722) (%)	Others (N=9,084)(%)	χ² (degrees of freedom)	p-Value
Disposition of the Patient								
Home	44.9 (91,236)	47.86 (20,013)	58.09 (22,918)	50.61 (5,596)	57.37 (1,562)	52.03 (4,728)	χ²(30) = 4.9 x 10^3^	<0.001
Short-Term Hospital	2.76 (5,612)	2.36 (987)	2.42 (955)	2.32 (256)	3.09 (84)	2.60 (236)		
Skilled-Nursing Facility	22.93 (46,628)	20.21 (8,452)	12.76 (5,036)	14.59 (1,613)	15.56 (424)	17.47 (1,587)		
Home-Health	20.42 (41,515)	18.51 (7,741)	17.47 (6,895)	22.59 (2,496)	12.65 (344)	17.51 (1,591)		
Discharge Against Medical Advice	1.75 (3,557)	2.73 (1,142)	2.45 (967)	1.06 (117)	3.79 (103)	2.14 (194)		
Mortality	7.22 (14,674)	8.32 (3,479)	6.80 (2,684)	8.83 (977)	7.54 (205)	8.25 (749)	χ²(5) = 124.43	<0.001
Length of Stay (Days) ± SD	8 ± 9.6	9 ± 13.0	7 ± 11.1	8 ± 11.1	8 ± 10.1	9 ± 12.5	χ²(5) = 8.5 x 10^3^, F-value = 192.22	<0.001
Total Charge ($) ± SD	117,152 ± 194,689	133,789 ± 242,825	147,987 ± 281,364	162,031 ± 281,631	102,711 ± 168,262	178,797 ± 343,534	χ²(5) = 1.9 x 10^4^, F-value = 312.50	<0.001

Multivariate logistic regression analysis was also performed after adjusting for confounders and White as the reference group. Compared to White patients, African American (adjusted odds ratio (AOR): 0.64 (95% CI: 0.61-0.66), p < 0.001), Hispanic (AOR: 0.65 (0.63-0.67), p < 0.001), and Native American patients (AOR: 0.49 (0.83-0.95), p < 0.001) were significantly less likely to be admitted electively for ITP. Asian or Pacific Islander patients showed no significant difference in elective admission rates compared to White patients (AOR: 1.03 (0.97-1.09), p = 0.29). African American patients had a significantly higher likelihood of in-hospital mortality compared to White patients, with an AOR of 1.20 (95% CI: 1.15-1.25, p = 0.02). Asian or Pacific Islander (AOR: 1.12 (1.05-1.20), p < 0.001) and Native American (AOR: 1.16 (1.03-1.25), p < 0.001) patients also demonstrated significantly increased odds of mortality. In contrast, Hispanic patients showed no significant difference in mortality risk compared to White patients (AOR: 1.00 (0.96-1.05), p = 0.70). The multivariate regression analysis affecting racial inequities in admission and mortality of ITP patients is summarized in Table 3.

**Table 3:** Multivariate regression analysis affecting racial inequities in admission and mortality of ITP patients in the 2022 NIS AORs were calculated after controlling for potential confounders, including socio-demographic variables, hospital characteristics, the Charlson Comorbidity Index, and established cardiac risk factors such as tobacco use, hypertension, hyperlipidemia, diabetes, cannabis use, and obesity. AOR: Adjusted odds ratio; CI: Confidence interval; ITP: Immune thrombocytopenia; NIS: National Inpatient Sample

Race	Elective vs. Non-Elective Admission AOR* (95% CI, p-value)	Mortality AOR* (95% CI, p-value)
White (203,258)	Reference	Reference
African-American (41,808)	0.64 (0.61-0.66, <0.001)	1.2 (1.15-1.25, 0.02)
Hispanic (39,463)	0.65 (0.63-0.67, <0.001)	1.0 (.96-1.05, 0.70)
Asian or Pacific Islander (11,054)	1.03 (0.97-1.09, 0.29)	1.12 (1.05-1.20, <0.001)
Native American (2,722)	0.49 (0.83-.0.95, <0.001)	1.16 (1.03-1.25, <0.001)

In-hospital complications

The adjusted analysis revealed notable racial disparities in complications associated with ITP hospitalizations and compared to White patients, Hispanic (AOR 1.39; 95% CI: 1.32-1.46; p < 0.001) and Asian or Pacific Islander patients (AOR 1.73; 95% CI: 1.60-1.88; p < 0.001) had significantly higher odds of receiving platelet transfusions, while African American patients had slightly lower odds (AOR 0.94; 95% CI: 0.88-0.99; p = 0.04). No statistically significant differences were observed across racial groups for epistaxis. However, the risk of sepsis was elevated among African American (AOR 1.03; p = 0.02), Hispanic (AOR 1.10; p < 0.001), Asian or Pacific Islander (AOR 1.22; p < 0.001), and Native American patients (AOR 1.43; p < 0.001) compared to Whites. In terms of AKI, African American (AOR 1.24; p < 0.001) and Native American patients (AOR 1.15; p < 0.001) had higher odds, while Hispanic (AOR 0.95; p < 0.001) and Asian or Pacific Islander patients (AOR 0.92; p < 0.001) had significantly lower odds compared to their White counterparts. The AOR for ITP-related complications by race is demonstrated in Table 4.

**Table 4:** Multivariate regression analysis of racial differences in the occurrence of various complications associated with ITP patients in the 2022 NIS AORs were calculated after controlling for potential confounders, including socio-demographic variables, hospital characteristics, the Charlson Comorbidity Index, and established cardiac risk factors such as tobacco use, hypertension, hyperlipidemia, diabetes, cannabis use, and obesity. AOR: Adjusted odds ratio; CI: Confidence interval; AKI: Acute kidney injury; ITP: Immune thrombocytopenia; NIS: National Inpatient Sample

Complications	White	African American AOR* (95% CI, p-value)	Hispanics AOR* (95% CI, p-value)	Asian or Pacific Islander AOR* (95% CI, p-value)	Native American AOR* (95% CI, p-value)
Platelet Transfusions	Reference	0.94 (0.88- 0.99, 0.04)	1.39 (1.32-1.46, <0.001)	1.73 (1.60-1.88, <0.001)	0.80 (0.64-1.01, 0.06)
Epistaxis	Reference	0.95 (0.86-1.05, 0.38)	1.08 (0.98-1.19, 0.09)	1.08 (0.91-1.28, .32)	0.93 (0.67-1.31, 0.71)
Sepsis	Reference	1.03 (1.00- 1.07, 0.02)	1.10 (1.06- 1.14, <0.001)	1.22 (1.16-1.30, <0.001)	1.43 (1.27-1.59, <0.001)
AKI	Reference	1.24 (1.21-1.27, <0.001)	0.95 (0.93-0.98, <0.001)	0.92 (0.88-0.96, <0.001)	1.15 (1.06-1.26, <0.001)

## Discussion

This retrospective analysis highlights racial disparities among patients hospitalized with ITP in the United States, as evidenced by the 2022 NIS data study. The investigation reveals that hospitalization patterns, clinical outcomes, and healthcare utilization vary significantly across distinct racial groups. Two prominent incidence peaks for ITP are early childhood and late adulthood. In children, the mean age is six years, and in adults, the mean age is around 65 years [7,8]. Our study's mean age of ITP hospitalization was 62 years, whereas prior studies had a mean age of 65 years [8]. Our analysis suggests that ITP-related hospitalizations occur at an older age among White and Asian populations. In contrast, minority groups, particularly Native Americans and Hispanics, tend to be hospitalized with ITP at younger ages. This age disparity may reflect underlying differences in disease onset, healthcare access, socio-economic factors, or comorbid conditions across racial groups.

The annual incidence of ITP in the United States is approximately 3.3 per 100,000 individuals [9]. While previous literature reports a roughly equal prevalence of ITP among elderly men and women, our analysis demonstrated a higher proportion of male hospitalizations (56.35%) compared to females (43.65%) [10,11]. This male predominance was consistently observed across all racial groups, suggesting potential differences in disease severity, healthcare utilization, or reporting patterns that warrant further investigation. Prior studies have noted that it affects people of all racial and ethnic backgrounds, although precise incidence rates by group remain unclear. In our analysis of the 2022 NIS data, ITP hospitalizations were most prevalent among White patients, followed by Hispanic and African American populations. The mortality rates of ITP among different race groups in the United States show a decrease in the mortality over the past decade, exclusively among white patients, with no significant reduction in mortality observed among Black or Hispanic patients [6]. However, our analysis of ITP hospitalization in 2022 found that the mortality rate was higher in Asian or Pacific Islander individuals (8.83%), followed by African American (8.32%) and those categorized as Other (8.25%). Mortality rates were slightly lower in Native American (7.54%) and White (7.22%) patients, while Hispanic patients had the lowest rate at 6.80%. These findings highlight persistent disparities in ITP outcomes and raise essential questions about underlying systemic factors across various racial groups.

Various socio-economic and systemic factors significantly contributed to the disparities among racial groups [12]. Insurance status, income level, and access to post-acute care are critical in shaping health trajectories. Previous literature reports that Medicaid and uninsured patients experience worse patient safety indicators and adverse events during hospitalization compared to those with private insurance [13]. Our analysis reveals pronounced racial disparities in both insurance coverage and geographic distribution among patients hospitalized with ITP, reflecting the broader influence of social determinants on disease management and outcomes. White and Asian patients were predominantly covered by Medicare, likely due to older age at presentation. In contrast, Native American, Hispanic, and African American patients exhibited higher rates of Medicaid coverage and self-pay status, indicating increased socio-economic vulnerability and potential barriers to accessing optimal care. These findings are consistent with prior ITP studies demonstrating that insurance status significantly impacts access to advanced therapies, such as thrombopoietin receptor agonists and specialist consultations, ultimately influencing clinical outcomes [14]. These findings highlight the urgent need for targeted interventions that address social determinants of health and enhance post-hospital care, particularly for underserved minority populations. Furthermore, they underscore the importance of including diverse racial and ethnic groups in clinical research and trials to inform equitable treatment guidelines.

The literature indicates an overall increase in epistaxis rates among ITP patients from 2010 to 2019 [15]. However, our analysis reveals no statistically significant racial disparities in epistaxis incidence, suggesting that this trend may be uniformly distributed across different races [6,15]. Our study highlights substantial racial disparities in complications associated with ITP hospitalizations in the United States. Hispanic and Asian or Pacific Islander patients had markedly higher odds of receiving platelet transfusions. This is consistent with prior evidence associating thrombocytopenia with worse outcomes and increased transfusion requirements [16]. Sepsis and AKI were more prevalent among African American and Native American patients, underscoring the impact of systemic and clinical risk factors [17]. These disparities emphasize the urgent need for equitable care strategies, improved inpatient monitoring, and increased representation of minority populations in ITP-related clinical research. Addressing social determinants of health and ensuring culturally sensitive care delivery are critical steps toward mitigating these inequities.

This study has several limitations inherent to the use of the NIS database. The database lacks granular patient-level details, such as medication use and laboratory values, which may impact the outcome of ITP hospitalizations [18]. Additionally, the reliance on administrative coding introduces the possibility of misclassification or coding errors, which could affect the accuracy of diagnoses and complication rates. The study's cross-sectional design further restricts causal inference; we can only describe associations rather than establish definitive cause-and-effect relationships between race, socio-economic status, and patient outcomes [18,19]. The NIS database includes only hospitalized patients within the United States, limiting the generalizability of our findings to outpatient populations or international settings [18,19].

## Conclusions

In conclusion, this study reveals significant racial and socio-economic disparities in hospitalization outcomes among patients with ITP. Minority populations, particularly Asian or Pacific Islander, African American, and Native American patients, experienced higher mortality rates and increased risk of complications such as sepsis and AKI compared to White patients. Additionally, disparities in age at presentation, length of stay, hospital charges, and post-discharge care further underscore the unequal burden of disease among these groups. These findings highlight the urgent need for targeted interventions, inclusive clinical research, and policy reforms to address social determinants of health and ensure equitable care and outcomes for all individuals affected by ITP.
